# Genotype Distribution Change After Human Papillomavirus Vaccination in Two Autonomous Communities in Spain

**DOI:** 10.3389/fcimb.2021.633162

**Published:** 2021-09-22

**Authors:** Javier Freire-Salinas, Rafael Benito, Ainara Azueta, Joaquina Gil, Claudia Mendoza, Montserrat Nicolás, Pilar García-Berbel, Sonia Algarate, Javier Gómez-Román

**Affiliations:** ^1^Servicio de Anatomía Patológica, Hospital Universitario Marqués de Valdecilla, IDIVAL, Universidad de Cantabria, Santander, Spain; ^2^Servicio de Microbiología, Hospital Clínico Universitario Lozano Blesa, Zaragoza, Spain

**Keywords:** human papillomavirus, vaccination, genotype, HPV–infection–prevalence–Europe–risk factors, change

## Abstract

**Context:**

It has been more than 10 years since the human papillomavirus (HPV) vaccination program was initiated in most advanced countries. Thus, it seems necessary to change the uterine cervical cancer screening strategy. Molecular-based tests are considered essential in this scenario.

**Objective:**

We aimed to review the distribution of the HPV genotypes after the introduction of the vaccination program with Cervarix® and Gardasil 4® in two autonomous communities in Spain, looking for possible changes in distribution and the occurrence of a herd effect.

**Design:**

A cross-sectional study was performed in 45,362 samples that were processed in the Cantabria and Aragon communities during the period from 2002 to 2016. We compared the genotype distribution before and after the vaccination program was initiated.

**Results:**

Genotypes HPV6 and HPV11 have decreased significantly after the introduction of the vaccine. HPV16 has had a decrease, but not a significant one in the statistical analysis. However, HPV31, HPV52, and HPV45 have increased in percentage. A replacement phenomenon with other genotypes not included in the vaccine has been observed in our population.

**Conclusions:**

Continued surveillance is needed to provide further indication of any changes over time in the genotypes in circulation. This will be facilitated by monitoring the genotyping results from the new model of cervical screening using primary HPV DNA testing.

## Introduction

Human papillomavirus (HPV) is responsible for one of the most common sexually transmitted infections in the world (WHO, 2016). Its importance lies in its oncogenic potential, and it is recognized as a type C agent within the classification of the World Health Organization (Bouvard et al., 2009).

In the field of primary attention care, there are two complementary developments that have changed the cervical cancer prevention protocols in the Western world: firstly, the implementation of vaccination programs in 2007 in 65 countries (WHO, 2013) and, secondly, the replacement of cytology and Pap staining-based preventive tests with molecular HPV diagnostic techniques using cytology as a second-line method for triaging patients (von Karsa et al., 2015).

In 2007, the Interterritorial Spanish Council of the National Health System (Consejo Interterritorial del Sistema Nacional de Salud) approved the systematic vaccination against HPV in a single cohort of girls aged from 11 to 14 years. Vaccination started in 2008 using the bivalent vaccine until 2014. From 2014 onwards, the quadrivalent vaccine is administered. In the Aragon community, vaccination was established in schools and that in Cantabria in health centers. In 2015, the overall coverage in Spain, for the complete vaccine regimen, was 77.8% (range = 65.6%–93.4%), while in Aragon it was 88.3% and in Cantabria 89.7%. The last data published from the Spanish Ministry of Health are from 2019; the complete regimen was 79.0% with a target population of 171,929 women. For the first dose, the Aragon coverage is 94.1% and that of Cantabria was 90.6% (Ministerio De Sanidad, Servicios Sociales E Igualdad - Profesionales - Vacunas Coberturas De Vacunación, 2017). Unfortunately, we only have the Cantabria data for the second dose, which is 75.5%.

The vaccination program was established with two commercially available inactivated vaccines: Cervarix® and Gardasil®. They were synthesized from non-infectious structural proteins obtained by genetic recombination. Cervarix® is active against the HPV16 and HPV18 genotypes and Gardasil® against the HPV6, HPV11, HPV16, and HPV18 genotypes, with some differences also in the adjuvant molecules (Harper and Demars, 2017). It is estimated that a 70% coverage must be reached in order to achieve herd immunity (Levin et al., 2015). A new nonavalent vaccine that protects against five additional high-risk HPV genotypes (HPV31, HPV33, HPV45, HPV52, and HPV58) has been recently developed (Gardasil9®).

While the effectiveness is not discussed in terms of the genotypes included in the vaccines, a number of different questions are raised about the cross-protective effects between the genotypes not included in the vaccine, a possible replacement of genotypes (Martcheva et al., 2008); a generalized herd-protective effect in the entire population, even in the unvaccinated; and the role of multiple infections, because women are still exposed to the other high-risk HPV genotypes that cause 30% of cervical cancers and oropharyngeal neoplasms and there is a risk of competition between genotypes to occupy the space left by those included in the vaccine (Yusupov et al., 2019).

The importance of HPV vaccination has increased because of the high prevalence of HPV-induced oropharyngeal cancers. In fact, this location has superseded the uterine cervix as the most prevalent site of papillomavirus-related neoplasms (Berman and Schiller, 2017).

Therefore, our aim in this study was to review the distribution of the HPV genotypes after the introduction of the vaccination program in two autonomous communities in Spain: Cantabria and Aragon. In both communities, HPV genotyping was performed as a routine molecular technique. Data of the genotyping prevalence from the previous years before the start of the vaccination (pre-vaccination) were compared with those from the years after implementation of the vaccination program (post-vaccination).

## Material and Methods

Our study populations before and after implementation of the HPV vaccine were recruited from the same geographic locations and category of service provider in order to minimize changes in the HPV-risk-related characteristics between the two periods.

A repeat cross-sectional study was performed in 45,362 samples that were processed in both communities during the period from January 2002 to May 2016. Of these, 17,472 were in Hospital Lozano Blesa (Aragón) and 27,890 were in Hospital Marques de Valdecilla (Cantabria). The aim of the study was to compare the prevalence of HPV along the years of opportunistic screening.

Specifically, we compared the HPV prevalence in the two populations by assessing the differences in any one of the 35 HPV genotypes tested (see below). Three HPV types that are genetically related to the vaccine-targeted HPV types—i.e., HPV45 (related to HPV18) and HPV31 and HPV33 (related to HPV16)—against which cross-protective antibodies have been detected in response to HPV vaccination, were the most closely studied. We also report prevalence individually for HPV52 and HPV58, which are also, but less closely, related to HPV16. We performed a *t*-test to find out whether there are remarkable differences between the averages pre- and post-vaccination, i.e., whether those differences may have happened by chance or not. A more complex statistical analysis was not performed because of the lack of enough data associated with the HPV results.

All the data were anonymized and no relationship with clinical records was obtained. Approval from the Ethics Committee for Clinical Investigation was obtained.

From an analytical point of view, cervical swabs were analyzed with a CLART HPV2 and HPV4 genotyping test (Genomica S.A.U., Madrid, Spain), which is based on PCR followed by low-density microarray for the determination of genotypes from specific HPV L1 fragments from 35 individual HPV genotypes (HPV6, HPV11, HPV16, HPV18, HPV26, HPV31, HPV33, HPV35, HPV39, HPV40, HPV42–45, HPV51–54, HPV56, HPV58, HPV59, HPV61, HPV62, HPV66, HPV68, HPV70–73, HPV81–85, and HPV89).

A sample collected with the cervical swab was resuspended in 1 ml saline solution immediately before processing. The resuspension was vortexed thoroughly for 1 min. DNA extraction was done using an automatic extractor following the manufacturer’s instructions: COBAS AmpliPrep (Roche Diagnostics, Mannheim, Germany) was used in Lozano Blesa Hospital and QIAcube (Qiagen, Hilden, Germany) was used in Marqués de Valdecilla Hospital.

Of the extracted DNA, 5 μl was used for PCR amplification. Amplification was performed on Eppendorf Mastercycler EP gradient S (Eppendorg AG, Hamburg, Germany) and Veriti (Applied Biosystems, Waltham, MA, USA). Automatic visualization was performed using Autoclart^®^ (Genomica S.A.U., Madrid, Spain) with 5 μl of the denatured PCR products. The precipitate was analyzed on a Clinical Array Reader (Genomica S.A.U., Madrid, Spain) with the CLART Human Papillomavirus 2 and 4 software.

The results were expressed in absolute values and in median and averages of positive HPV tests related to the total of tests analyzed.

## Results

There were 27,890 patients from the Cantabria autonomous community and 17,472 patients from the Aragon autonomous community evaluated for the period between January 2002 and May 2016. A total of 3,395 HPV-positive women were studied from the pre-vaccination period and 17,648 from the post-vaccination period. The average age of these HPV-positive women was 37.3 years, with a median of 36.3 years (pre-vaccination: mean = 37.8 years, age range = 14–69 years; post-vaccination: mean = 37.2 years, age range = 14–69 years). The proportion of patients with a positive test for HPV infection was 46.38% (21,043 patients).

Upon verifying the distribution of the HPV genotypes over the years, it was observed that most of the 35 genotypes analyzed in the PCR system did not show temporal differences (**Supplementary Table S1**).

**Figure 1:**
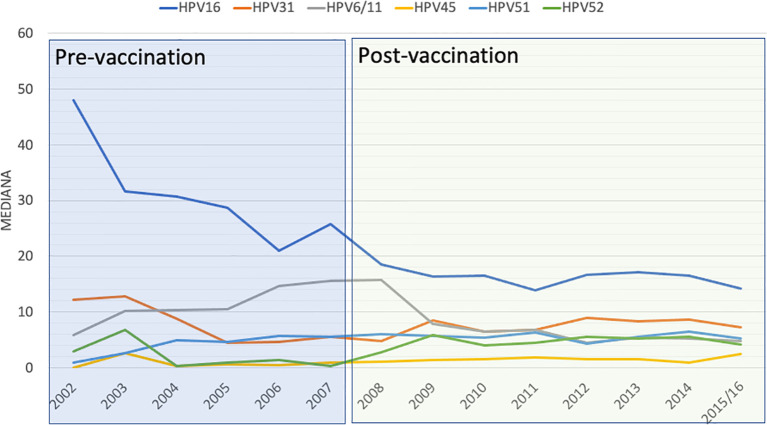
Distribution of the different human papillomavirus (HPV) genotypes along the different years of study.

By analyzing more closely the data of the genotypes included in the vaccine, we observed that the HPV16 genotype showed an important decrease over the years, starting from the date of the initiation of vaccination, although statistical analysis was not significant (*p* = 0.069) (**Figure 1**). The effect was also observed for the low-risk genotypes HPV6 and HPV11, although in this case the differences were significant (*p* = 0.007) (**Figure 1** and **Table 1**). However, the HPV18 genotype showed no significant temporal changes similar to those of HPV33.

**Table 1:** Averages of the main human papillomavirus (HPV) genotypes in the pre- and post-vaccination periods.

Genotype	Pre-vaccination^a^	Post-vaccination^a^	*p*-value
HPV6^b^	324 (7.55)	799 (4.40)	<0.01
HPV11^b^	125 (3.87)	407 (1.68)	<0.01
HPV16	927 (28.73)	2,816 (16.57)	>0.05
HPV18	183 (4.15)	594 (3.08)	>0.05
HPV31	219 (5.55)	1,308 (7.81)	<0.01
HPV45	28 (0.67)	285 (1.46)	0.01
HPV51	165 (4.68)	987 (5.64)	>0.01
HPV52	43 (1.38)	811 (4.83)	<0.01

Total pre-vaccination cases analyzed: 3,395. Total post-vaccination cases analyzed: 17,648.

a Values shown are the total number and averages (in parenthesis).

b Low-risk genotypes.

The other genotypes that showed significant changes in distribution were HPV31, which underwent a significant increase (*p* = 0.006), and HPV45 (*p* = 0.01) and HPV52 (*p* = 0.001) (**Figure 1** and **Table 1**).

The rest of the less prevalent genotypes showed no evident changes in average values in the analysis.

## Discussion

The decision to introduce HPV vaccination into the national immunization program in Spain was made in October 2007, recommending the vaccination of girls aged between 11 and 14 years with three doses of the HPV vaccine. The two aforementioned regions introduced HPV vaccination in the beginning of 2008. Aragon administered the vaccines in schools and Cantabria in health centers. The coverage in both cases exceeded 70% of the population according to official data (Ministerio De Sanidad, Servicios Sociales E Igualdad - Profesionales - Vacunas Coberturas De Vacunación., 2017), which means that the protection is adequate and that the study data are comparable because they exceeded the optimum cost-effectiveness balance limit.

HPV has different oncogenic potentials depending on the genotype. Monitoring evolution in the distribution of genotypes is therefore critical in establishing early detection policies based on molecular techniques that are progressively replacing those based on cytology.

Vaccines directed against infectious agents that have different serotypes and limited coverage against some of them may show long-term challenges. For example, a heptavalent vaccine against pneumococcus was established in the late 1990s, and due to genomic instability, an increase in non-vaccine serotypes was observed in a short time. This same phenomenon was observed with *Bordetella pertussis* (Singleton et al., 2007). However, this is a viral disease and the introduction of the nonavalent vaccine and cross-reactivity with more genotypes included could change this limited coverage.

Nevertheless, HPV is very stable with a mutation rate of one base per 10,000 years (Zur Hausen, 1996), which is why the occurrence of resistance mutations is extremely rare. Therefore, problems may arise due to the competition between genotypes in order to achieve a successful infection, when cross-protection for the genotypes not included in the vaccine is not available or is questionable.

We have observed a decrease in prevalence among the HPV genotypes included in the vaccine (HPV16, HPV6, and HPV11), although this was non-significant in the case of HPV16. It is important to note that the coverage of the second dose is lower, at least in Cantabria. We have not observed the same effect in the HPV18 genotype, perhaps due to the low prevalence of this virus in the Spanish population. These data are comparable to those obtained in other series (Tabrizi et al., 2014; Dillner et al., 2018).

As for data referring to the possible replacement of HPV31 and HPV45, it should be noted that an Australian study also found increases in the prevalence of both viruses in the non-vaccinated population (Tabrizi et al., 2014), although these were only statistically significant in the case of HPV45, probably due to the scarcity (low number) of cases in its publication. Thus, it appears that the replacement and occupancy of a niche of infection left by the vaccine genotypes HPV31 and HPV45 is real. Given the high oncogenicity of HPV45, these data are relevant for the inclusion of genotyping in the protocols for early detection and for moving to nonavalent vaccination. There are, however, some follow-up papers identifying that this phenomenon is more likely due to the detection assays used, with data suggesting that some HPV genotyping assays preferentially amplify HPV16 and HPV18 over HPV31, HPV33, HPV51, and HPV59, with the preserved increase a false finding (Cornall et al., 2015). Our study used the same assay before and after the vaccine introduction, and we confirm these data as real and not induced for methodological aspects. Other recent papers have shown significant increases in HPV genotypes unrelated to nonavalent vaccination (Gardella et al., 2021).

According to the principles of ecology (Gause, 1934), several species cannot compete stably for the same niche. This principle, applied to viral infection, would indicate that, if there is competition between more than two HPV genotypes for the occupancy of a niche during a natural infection, elimination of one type may result to an increase in other genotypes. Multi-infection is a frequent phenomenon that reaches 32%–47% of all infections in our experience (data not published). Faced with the question of what will happen if one of them disappears and the others remain, on the possible oncogenic role of the rest, we do not have an answer. Although in our study we have shown an increase in certain non-vaccine viruses that could even be artefactual, it might be that they corresponded to undetected genotypes and they surface when the main genotypes disappear.

On the other hand, there is what is called the “herd effect,” which provides indirect protection for those who have not been vaccinated because of a reduced prevalence of HPV in the population. In this sense, we have detected a decrease in the genotypes included in the vaccine. Therefore, it might be appropriate to consider this effect when establishing policies for early detection in the non-vaccinated population.

The herd effect that has already begun to be detected in some populations (Tabrizi et al., 2014) may have been observed in our study showing a decrease in the overall prevalence of the HPV genotypes included in the vaccine.

A final topic that we consider necessary to discuss is the technique used for the molecular diagnosis of HPV infection. All of the data obtained in our study and all the questions posed about the replacement of genotypes and herd effect would not have been determined and answered if not for the use of a genotyping technique. In different countries, there is a trend toward the use of techniques based on PCR and genotyping for diagnosis (Zhao et al., 2016). In fact, in another study from Spain, the cumulative data showed that multiple infections were diagnosed in 22% of the cases and that the HPV genotype distribution is different from that of previously published data when multiple types are included in the screening (Gomez-Roman et al., 2009).

Finally, the intrinsic biological malignant potential of the different genotypes is another influencing factor to favor genotyping. In fact, it is well known that the risk of the HPV16 or the HPV18 genotype is higher than that of the rest (Schiffman et al., 2015).

We acknowledge that our study has several limitations. Firstly, we have no access to the clinical data of all the patients as we do not know the vaccination status in all cases. Secondly, our only aim was to present a description of real-world data in order to best decide on appropriate strategies for vaccination and screening.

In conclusion, continued surveillance is needed to provide further indication of any changes over time in genotypes in circulation. This will be facilitated by the monitoring of the genotyping results from the new model of cervical screening using primary HPV DNA testing.

## Data Availability Statement

The original contributions presented in the study are included in the article/**Supplementary Material**. Further inquiries can be directed to the corresponding author.

## Ethics Statement

The studies involving human participants were reviewed and approved by Comité de Ética de la Investigación con Medicamentos de Cantabria. Written informed consent for participation was not required for this study in accordance with the national legislation and the institutional requirements.

## Author Contributions

JF-S: Design, writing, and technical support. RB: Design and writing. AA: Writing and technical support. JG: Technical support. CM: Technical support. MN: Technical support. PG-B: Technical support and statistical analysis. SA: Technical support. JG-R: Design and writing. All authors contributed to the article and approved the submitted version.

## Conflict of Interest

The authors declare that the research was conducted in the absence of any commercial or financial relationships that could be construed as a potential conflict of interest.

## Publisher’s Note

All claims expressed in this article are solely those of the authors and do not necessarily represent those of their affiliated organizations, or those of the publisher, the editors and the reviewers. Any product that may be evaluated in this article, or claim that may be made by its manufacturer, is not guaranteed or endorsed by the publisher.
